# The mid-term effects of carotid endarterectomy on cognition and regional neural activity analyzed with the amplitude of low frequency fluctuations technique

**DOI:** 10.1007/s00234-021-02815-7

**Published:** 2021-09-25

**Authors:** Michele Porcu, Luigi Cocco, Riccardo Cau, Jasjit S. Suri, Lorenzo Mannelli, Qi Yang, Giovanni Defazio, Max Wintermark, Luca Saba

**Affiliations:** 1 Department of Radiology, AOU Cagliari, University of Cagliari, Cagliari, Italy; 2 Stroke Diagnosis and Monitoring Division, AtheroPoint™, Roseville, CA USA; 3 Department of Radiology, IRCCS SDN, Naples, Italy; 4 Xuanwu Hospital, Capital Medical University, No.45 Changchun Street, Xicheng District, Beijing, China; 5 Department of Neurology, University of Cagliari, Cagliari, Italy; 6 Department of Neuroradiology, Stanford University, Stanford, CA USA

**Keywords:** ALLF, Carotid endarterectomy, Cognition

## Abstract

**Purpose:**

The study aims to evaluate the mid-term effects of carotid endarterectomy (CEA) on cognition and resting-state functional magnetic resonance imaging (rs-fMRI) using the Amplitude of Low Frequency Fluctuations (ALFF) technique.

**Methods:**

In this observational study, patients eligible for CEA were prospectively included. On the same day, within 1 week of the CEA procedure performed and 12 months after the CEA procedure, all patients underwent (i) an MRI examination for rs-fMRI analysis and (ii) a cognitive evaluation using the Italian version of the Mini-Mental State Examination (MMSE) corrected for age and schooling. Pre-CEA and post-CEA MMSE scores were evaluated using paired sample *t*-tests, adopting a *p*-value < 0.05 as statistical threshold. The ALFF technique was used for analyzing the differences between pre-CEA and post-CEA rs-fMRI scans in terms of regional neural activation. This was accomplished by applying non-parametric statistics based on randomization/permutation for cluster-level inferences, adopting a cluster-mass *p*-value corrected for false discovery < 0.05 for cluster threshold, and a *p*-uncorrected < 0.01 for the voxel threshold.

**Results:**

Twenty asymptomatic patients were enrolled. The mean MMSE score resulted improved following CEA procedure (*p*-value = 0.001). The ALFF analysis identified a single cluster of 6260 voxels of increased regional neural activity following CEA, and no cluster of reduced activity. The majority of voxels covered the right precentral gyrus, the right middle frontal gyrus, and the anterior division of the cingulate gyrus.

**Conclusion:**

Mid-term cognitive improvements observed after CEA are associated to increased regional neural activity of several cerebral regions.

**Supplementary Information:**

The online version contains supplementary material available at 10.1007/s00234-021-02815-7.

## Introduction

Ischemic stroke represents one of the main causes of morbidity and mortality worldwide. Approximately 800,000 strokes occur each year in the United States, and 87% of the cases are due to ischemic pathogenesis [1, 2]. Extracranial carotid artery stenosis is considered an important risk factor for ischemic stroke and transient ischemic attacks (TIAs) [3]. In particular, according to the European Society of Cardiology (ESC) guidelines on peripheral artery disease, 10–15% of all strokes are caused by thromboembolism from patients with internal carotid artery (ICA) stenosis ranging from 50 to 99% [4]. Further, several neuropsychological studies also evidenced that ICA stenosis is associated with impairment in neurocognitive functions [5, 6].

According to the current ESC guidelines [4], carotid revascularization by carotid endarterectomy (CEA) and carotid artery stenting (CAS) represents the treatment of choice for preventing stroke in patients with asymptomatic patients with ICA stenosis 60–99% and symptomatic patients with 50–99% stenosis. Even if it has been and still it remains a matter of debate whether carotid revascularization is associated or not with improvement of neurocognitive deficits [7], recent studies such as the one by Carta MG et al. [8] and by Whooley JL et al. [9] suggest that non-complicated CAS and CEA are associated with improvements in neurocognitive function, in particular in younger patients with worse neuropsychological performances [10]. Although various hypotheses have been formulated, the biological mechanisms underlying these changes still remain largely unknown.

Rs-fMRI represent a useful tool for the analysis of neural activity and brain networking [11]. Using this technique, it has been possible to better understand the cerebral networking impairments underlying the cognitive deficits observed in patients with ICA stenosis [12]. From a clinical point of view, this method of analysis could also give useful information for understanding the effects of carotid revascularization on the cerebral mechanisms underlying the changes observed on the higher neurological functions. For example, a recent study by Wang T et al. [13] showed that CAS is associated with improvements in cognition and memory, observing changes of the regional neural activity in rs-fMRI through the Amplitude of Low Frequency Fluctuations (ALFF) technique. ALFF is a rs-fMRI technique widely used in research for the analysis of regional neural activity, and it measures the total power of the blood oxygen level dependent (BOLD) signal in the low-frequency range [11, 14], and it is characterized by high temporal stability and test–retest reliability [11, 15, 16].

Based on these previous studies, we hypothesized that the cognitive improvements observed following CEA procedure are accompanied by changes of neural activity in analogy to what observed for CAS. We tested this hypothesis by designing an observational prospective study in which we analyzed the mid-term (12 months) effects of CEA on cognitive performances and on neural activity through the ALFF technique in a cohort of asymptomatic patients eligible for CEA.

### Institutional review board approval

Institutional review board approval was approved by local ethical committee.

## Materials and methods

### Study population

The institutional review board approved the study, in accordance with the ethical standards as laid down in the 1964 Declaration of Helsinki and its later amendments or comparable ethical standards. Consecutive patients with asymptomatic mono-lateral ICA stenosis eligible for CEA according to the European Society of Cardiology (ESC) guidelines [4] were enrolled at our university hospital in the period between September 2018 and December 2019; in particular, all the patients suffered of a unilateral stenosis ≥ 70% according to the North American Symptomatic Carotid Endarterectomy Trial (NASCET) index [17]. In analogy to previous studies [18, 19], patients with at least one of the following exclusion criteria were excluded from the recruitment: (a) not right-handed dominant patients; (b) patients with medical history of severe systemic inherited or acquired disease (in particular symptomatic patients, i.e., patients with clinical history of amaurosis fugax, TIA and major stroke ipsilateral to the lesion [20], and patients with other severe psychiatric/neurological diseases), except cognitive dysfunction; (c) contraindications for MRI examinations, such as the presence of non-compatible metallic devices; (d) presence of functional disability (values ≥ 2 according to modified Rankin scale [21]); and (e) patients with significant incidental pathologies identified during the execution of the MRI scan.

All the patients gave their written informed consent before enrollment.

### Cognitive assessment and MRI examination

The week before the CEA procedure, in the same day, all the patients performed the Italian version of the Mini-Mental State Examination (MMSE) corrected for age and schooling [22, 23] in order to evaluate the cognitive performances, and an MRI scan.

In analogy to previous studies [18, 19], a baseline (pre-CEA) MRI scan was performed with a 1.5-T Philips “Achieva dStream” scanner (Philips, Best, Netherlands), with a 16-channel head coil. The dedicated MRI scan protocol for resting state MR analysis included the following two sequences: (a) structural isotropic 3D T1-weighted Turbo Field Echo (TFE) sequence (TE = 3.43 ms, TR = 7.5 ms, flip angle = 8°, slice thickness = 1 mm, spacing between slices = 1 mm) and (b) resting state functional T2-weighted Echo-Planar Imaging (EPI) sequence (TE = 50 ms, TR = 3000 ms, flip angle = 90°, slice thickness = 5 mm, matrix: 80 × 80, volumes acquired: 326). Prior to the examination, all the patients were carefully instructed by the radiologist to follow technologist’s instructions during MR examination; in particular it was recommended to keep the eyes closed without thinking of anything while in a fully relaxed state during the execution of the functional T2-weighted EPI sequence. The order of the sequences of the MRI protocol was the same for all the patients.

The follow-up (post-CEA) cognitive assessment and MRI examination, with the same MRI scanner and with the same modalities, were performed on the same day 12 months after CEA procedure.

### MMSE scores analysis

A Kolmogorov–Smirnov normality test (with Lilliefors correction) was performed for verifying the normal distribution of pre-CEA and post-CEA MMSE scores, assuming a *p*-value = 0.2 as lower bound of the true significance. Once the normal distribution was verified, the pre-CEA and post-CEA MMSE scores were compared with a paired sample *t*-test, adopting a *p*-value < 0.05 as statistical significance threshold. Both the Kolmogorov–Smirnov test and the paired sample *t*-test were calculated by using the SPSS 24 statistical package (SPSS Inc., Chicago, IL).

### fMRI analysis

The fMRI analysis was made on the Matlab platform vR2020b (Mathworks, Inc., CA, USA) with the CONN-fMRI fc toolbox v20b [24] based on the SPM 12 package (Wellcome Department of Imaging Neuroscience, London, UK; http://www.fil.ion.ucl.ac.uk/spm/).

Similarly to previous studies [18, 19], structural 3D T1-weighted TFE and functional T2-weighted EPI sequences were pre-processed according to the CONN’s default pipeline for volume-based analysis with the following steps: (a) functional realignment and unwarping, followed by slice-timing correction; (b) functional outlier detection with intermediate settings (97th percentile in normative sample in functional outlier detection system: global-signal *z*-value threshold = 5 standard deviations; subject-motion threshold = 0.9 mm);(c) functional and structural direct segmentation of grey matter, white matter and cerebrospinal fluid, and subsequent normalization to Montreal Neurological Institute (MNI) exploiting the default tissue probability maps (structural target resolution = 1 mm; functional target resolution = 2 mm), and (d) functional smoothing with 8-mm full width half maximum Gaussian kernel filter.

The first 5 volumes of T2-weighted EPI sequence were excluded from analysis in order to limit the potential bias derived by the attainment of the steady state magnetization [25]. Subsequently, the following denoising steps were applied in order to minimize the residual non-neural variability of functional data: (a) linear regression of potential confounding effects, including BOLD signals recorded in cerebrospinal fluid and white matter [18, 19, 26], estimated subject-motion specifications, and identified outlier scans for the “scrubbing” procedure [27] and (b) temporal band-pass filtering (0.008 to 0.09 Hz) for decreasing noise effects and low-frequency drift [18, 19].

ALFF maps were created by computing for each individual voxel the root mean square of BOLD signal in the low frequency range (0.008 to 0.09 Hz) [14, 28]. The General Linear Model (GLM) was applied in the second-level group analysis for identifying statistically significant changes of BOLD signal following CEA by exploiting a paired *t*-test, using pre-CEA and post-CEA scans for between-condition contrast. Non-parametric statistics based on randomization/permutation was used for cluster-level inferences using 1000 permutation iterations of the original data, adopting a cluster-mass *p*-value corrected for false discovery (cluster-mass p-FDR) < 0.05 for cluster threshold and a *p-*uncorrected (p-unc) < 0.01 for voxel threshold [28–31].

The mapping of brain regions was made by using the CONN’s default atlas; in particular the Harvard–Oxford atlas [32] was adopted for cortical and subcortical regions, and the Automated Anatomical Labeling (AAL) atlas [33] for the cerebellar regions (Supplementary table 1).

## Results

### Study population

The final study population consisted of 20 asymptomatic patients, 14 males and 6 females (overall mean age = 75.09; mean age female group = 73.33; mean age male group = 74.45). CEA procedures were performed in the right side in 11 patients, whereas on the left side in 9 cases. None of the patients met the exclusion criteria above mentioned; in particular no incidental pathologic findings were detected during the execution of the baseline MRI scan. No procedural or peri-procedural complications following CEA, and the clinical course between the baseline and the follow-up assessment was uneventful. The demographic data are reported in Table 1.

**Table 1:** Population study - Demographic data

Population study - Demographic data		
Number of patients	Males	14
	Females	6
	Overall	20
Mean age	Males	74.45
	Females	73.33
	Overall	75.09
Side of ICA stenosis treated with CEA	Right	11
	Left	9

### Cognitive assessment

**Fig. 1:**
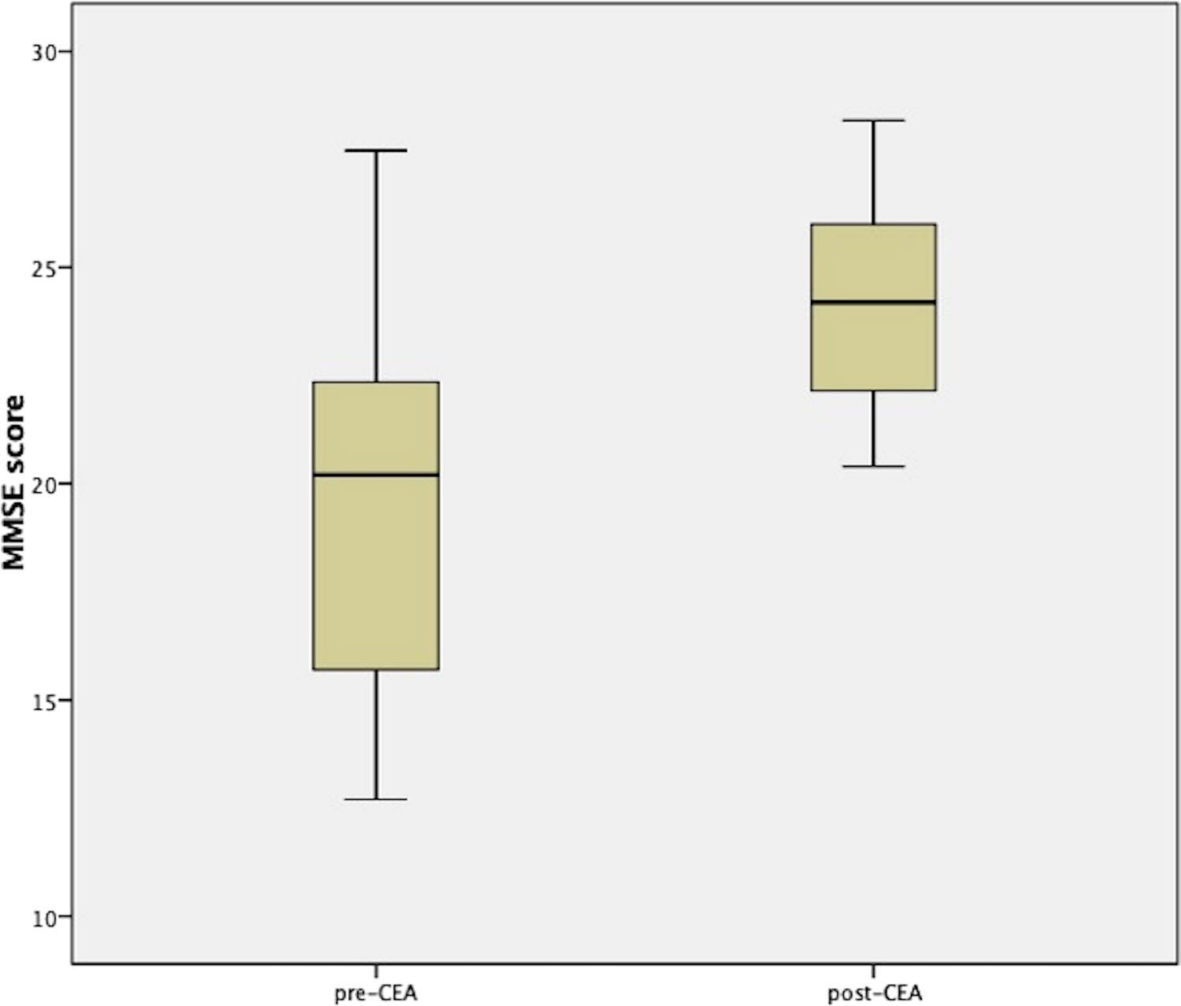
Box plots evidencing the differences between pre-CEA and post-CEA MMSE scores

Kolmogorov–Smirnov normality test (with Lilliefors correction) confirmed the normal distribution of both pre-CEA and post-CEA MMSE scores (*p*-value for pre-CEA MMSE scores = 0.111; *p*-value for post-CEA MMSE scores = 0.109). The paired sample *t*-test revealed statistically significant differences between the pre-CEA and the post-CEA MMSE scores (*p*-value = 0.001): in particular, the mean pre-CEA MMSE score was 19.62 (minimum value = 12.7; maximum value = 27.7) and the mean post-CEA MMSE score was 24.17 (minimum value = 20.4; maximum value = 28.4). The full statistics of the paired sample *t*-test are reported in Tables 2 and 3 and in Fig. 1.

**Table 2:** Paired sample *t*-test — statistics of pre-CEA and post-CEA MMSE scores

Paired Sample t test — statistics		
	Pre-CEA	Post-CEA
Mean score	19.62	24.17
Number of samples	20	20
Standard deviation	4.4027	2.59982
Standard error of mean	0.98447	0.58134

**Table 3:** Paired sample *t*-test — pair differences between pre-CEA and post-CEA MMSE scores

Paired sample *t*-test — pair differences between pre-CEA and post-CEA MMSE scores		
Mean score improvement	4.55	
Standard deviation	3.27711	
Standard error of mean	0.73278	
95% confidence interval of the difference	Lower bound	6.08374
	Upper bound	3.01626
*t*-value	6.209	
Degrees of freedom	19	
*p*-value	< 0.001

### fMRI analysis

**Fig. 2:**
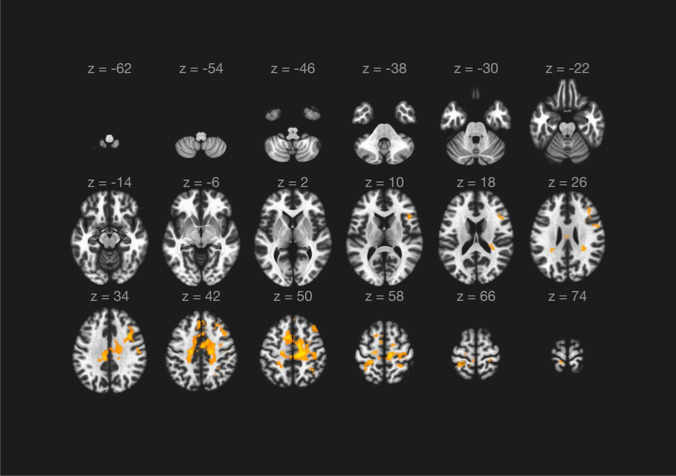
Results of the ALFF analysis (neurological orientation). The orangish areas represent areas of increased regional activation following carotid endarterectomy. The complete composition of the cluster of increased regional neural activity is reported in Table 4

**Fig. 3:**
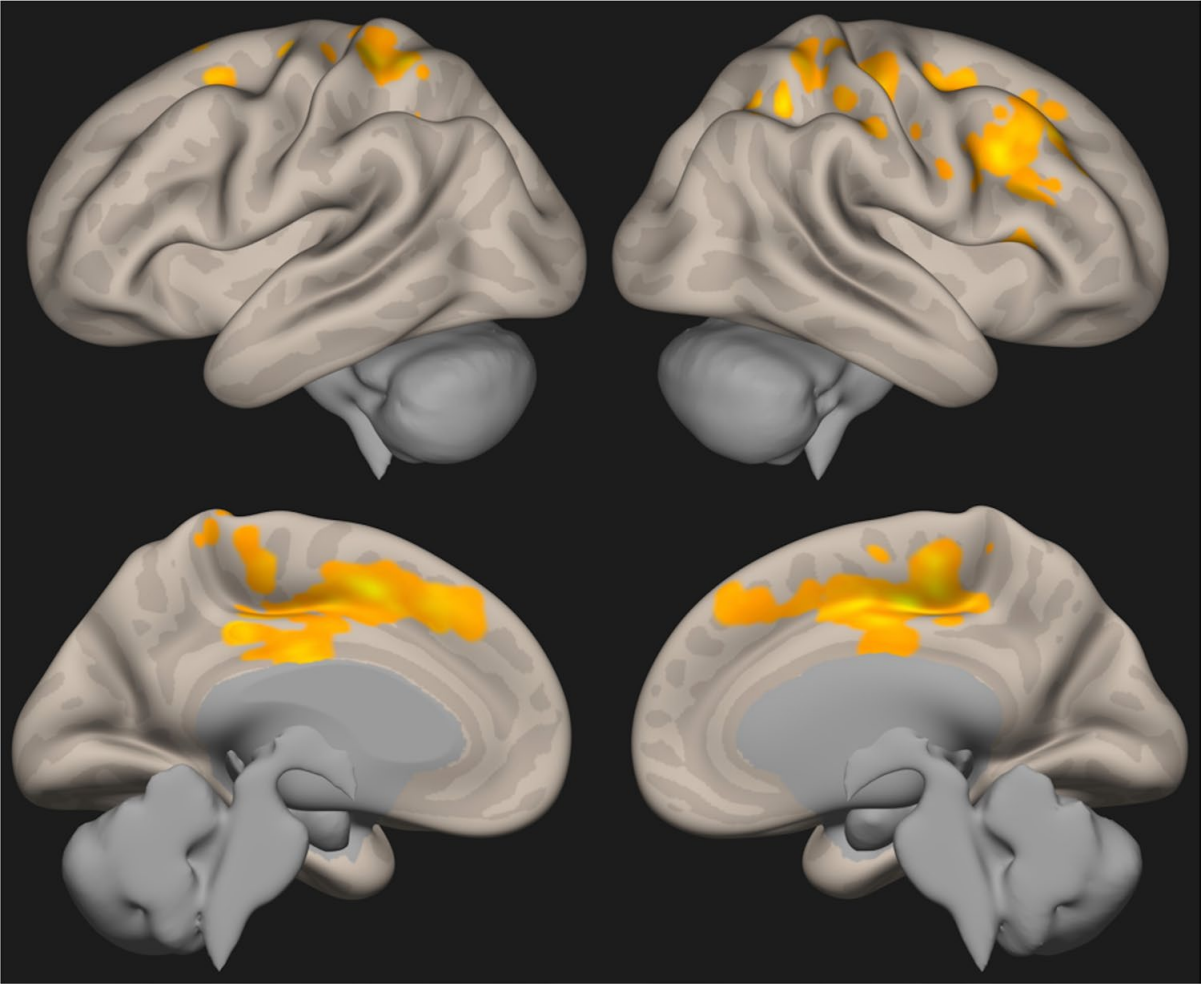
Results of the ALFF analysis (three-dimensional reconstruction). The orangish areas represent areas of increased regional activation following carotid endarterectomy. The complete composition of the cluster of increased regional neural activity is reported in Table 4

The quality control data of the study population following rs-fMRI preprocessing are reported in Supplementary table 2. The ALFF analysis evidenced the presence of a single cluster of voxels of increased regional neural activity following CEA procedure and no cluster of reduced activity. In particular, the cluster of increased activity consisted of 6260 voxels (cluster mass p-FDR = 0.004704). The majority of voxels identified in the analysis covered the right precentral gyrus (574 voxels, i.e., the 9% of the total amount), the middle frontal gyrus (457 voxels, i.e., the 7% of the total amount), and the anterior division of the cingulate gyrus (299 voxels, i.e., the 5% of the total amount). The complete statistics are reported in Table 4 and a visual representation is reported in Figs. 2 and 3.

**Table.4:** Results of ALFF analysis based on randomization/permutation method [29]* p-FWE* family wise error corrected *p*-value, *p-FDR p*-value corrected for false discovery rate, *p-unc p*-value uncorrected.

ALFF analysis								
*Cluster* *(x,y,z)*	*Cluster size (voxels)*	*Cluster size p-FWE*	*Cluster size p-FDR*	*Cluster size p-unc*	*Cluster mass*	*Cluster mass p-FWE*	*Cluster mass p-FDR*	*Cluster mass p-unc*
+ 14, − 16, + 46	6260	0.005000	0.003776	0.000026	72978.20	0.006000	0.004704	0.000032
*Voxels identified and relative brain areas*								
• 574 voxels (9%) covering 13% of atlas.PreCG r (precentral gyrus right) • 457 voxels (7%) covering 17% of atlas.MidFG r (middle frontal gyrus right) • 299 voxels (5%) covering 12% of atlas.AC (cingulate gyrus, anterior division) • 280 voxels (4%) covering 9% of atlas.PostCG r (postcentral gyrus right) • 273 voxels (4%) covering 7% of atlas.PostCG l (postcentral gyrus left) • 203 voxels (3%) covering 32% of atlas.SMA L(juxtapositional lobule cortex — formerly supplementary motor cortex — left) • 173 voxels (3%) covering 12% of atlas.SPL r (superior parietal lobule right) • 161 voxels (3%) covering 23% of atlas.SMA r (juxtapositional lobule cortex — formerly supplementary motor cortex — right) • 145 voxels (2%) covering 5% of atlas.SFG l (superior frontal gyrus left) • 138 voxels (2%) covering 3% of atlas.PreCG l (precentral gyrus left) • 132 voxels (2%) covering 10% of atlas.PaCiG l (paracingulate gyrus left) • 118 voxels (2%) covering 5% of atlas.PC (cingulate gyrus, posterior division) • 88 voxels (1%) covering 6% of atlas.SPL l (superior parietal lobule left) • 76 voxels (1%) covering 6% of atlas.PaCiG r (paracingulate gyrus right) • 59 voxels (1%) covering 2% of atlas.SFG r (superior frontal gyrus right) • 28 voxels (0%) covering 1% of atlas.sLOC r (lateral occipital cortex, superior division right) • 26 voxels (0%) covering 0% of atlas.FP r (frontal pole right) • 26 voxels (0%) covering 3% of atlas.aSMG r (supramarginal gyrus, anterior division right) • 21 voxels (0%) covering 3% of atlas.IFG oper r (inferior frontal gyrus, pars opercularis right) • 16 voxels (0%) covering 3% of atlas.IFG tri r (inferior frontal gyrus, pars triangularis right) • 8 voxels (0%) covering 3% of atlas.FO r (frontal operculum cortex right) • 4 voxels (0%) covering 0% of atlas.Precuneous (precuneous cortex) • 3 voxels (0%) covering 0% of atlas.AG r (angular gyrus right) • 1 voxels (0%) covering 0% of atlas.pSMG r (supramarginal gyrus, posterior division right) • 2951 voxels (47%) covering 1% of atlas.not-labeled

## Discussion

The role of CEA in stroke prevention has been extensively studied in literature [4], but less is known about the effects of this procedure on the higher neurological functions brain activity. The comprehension of the neural adaptive mechanisms following the CEA procedure could be of interest for understanding the possible role of this procedure not only in prevention of stroke, but also as a therapy for neurocognitive impairment in selected patients with ICA stenosis.

According to this idea, we explored in this study the impact of CEA on mid-term (12 months) cognitive performances and on regional neural activity changes with the ALFF method. Regarding the cognitive performances, we demonstrated that CEA was associated with statistically significant improvements in MMSE score, and the ALFF analysis evidenced increased ALFF signal in several areas of the brain, in particular in the right precentral gyrus, right middle frontal gyrus, and the anterior cingulate cortex (ACC). To the best of our knowledge, this is the first longitudinal study that used ALFF to investigate the mid-term arrangements in regional neural activity after CEA treatment. The ALFF technique is a functional segregation method of analysis of rs-fMRI data that allows to evaluate the regional neural activity, and this technique of analysis was considered fit for the aim of prospective research because of its high temporal stability and test–retest reliability [11, 15, 16], as we have already underlined in the introduction.

For better understanding these findings, it is useful to resume the principal scientific evidences derived from literature regarding the association between extracranial ICA stenosis, cognitive impairment, and anatomical and functional rearrangements of the brain, as well as the effects of CEA on cognition and brain activity evaluated by using the rs-fMRI technique.

Regarding the first point, the literature tends to confirm that extracranial ICA stenosis is associated with cognitive impairment [12]. The cognitive deficit observed in these patients could be attributed, at least partly, to the dysfunction of brain networking following a complex series of vascular rearrangements and structural changes of the brain [12]. In their research, Cheng HL et al. [34] compared a group of 17 patients with ≥ 70% asymptomatic unilateral ICA stenosis with 26 healthy controls utilizing a comprehensive neuropsychological battery and multimodality neuroimaging including diffusion tensor imaging (DTI) and rs-fMRI. The authors found for the first-time distinct patterns of network disruption that correlate with cognitive fragility in patients with asymptomatic ICA stenosis. In particular, the subjects with ICA stenosis showed reduced whole-brain fractional anisotropy at the DTI analysis, indicative of generalized white matter degeneration; further, at the rs-fMRI analysis they showed also regional specific disruption of default mode network (DMN), involved in memory and in recollection of prior experiences [35], and in the fronto-parietal network, a flexible hub for cognitive control, correlated with fluid intelligence [36]. In addition, a study conducted by Lin CJ et al. [37] supports the idea that cognitive decline in stroke-free individuals with severe ICA stenosis may arise from nonselective widespread disconnections of long-range, predominantly interhemispheric non-hippocampal pathways. Furthermore, connectivity measures may serve as both predictors for cases at risk and therapeutic targets for mitigating vascular cognitive impairment [37]. In this sense, it is noteworthy to mention the recent study by Wang T et al. [20], which postulated that asymptomatic patients with ICA stenosis had cognitive impairment in tests of executive function, psychomotor speed, and memory. This suggests that, although patients with carotid stenosis could be “asymptomatic” because of the absence of history of neurological deficits such as amaurosis fugax, TIA, or major stroke ipsilateral to the side of ICA stenosis [20], they may not be truly asymptomatic from a neurocognitive point of view. In particular, the prolonged cerebral hypoperfusion due to the presence of ICA stenosis could induce abnormalities in neuron electron activity and protein synthesis leading to cognitive impairment [12, 37]. These observations are sustained by the results of their study, in which asymptomatic patients with ICA stenosis and cognitive impairments, when compared to healthy controls, showed reduced cerebral blood flow in the left inferior frontal gyrus. This was measured with the pulsed arterial spin labeling technique, altered N-acetil-aspartate/creatinine ratio in the left hippocampus at the proton MRI spectroscopy, and reduced regional neural activity measured with the ALFF technique in the left and right supra medial frontal lobes associated with decreased connectivity to the posterior division of the cingulate cortex (PCC) in the anterior part of DMN [37].

Regarding the second point, both the carotid revascularization treatments, CEA and the CAS, are linked to long-term cognitive improvements [38]. Some studies tried to verify if there are differences between CEA and CAS effect on cognition; although carotid revascularization results in an overall improvement in cognitive function, there are no differences in the composite scores of five major cognitive domains between CEA and CAS [39]. Further, these evidences have been confirmed by a more recent study published in 2020 by Huang P et al. [40], which support the idea that CAS and CEA are effective in improving the cognitive function of patients with carotid stenosis, with no significant difference between them. From a functional point of view, it has been also demonstrated in previous studies that both CAS and CEA are associated to short-term changes in brain activity identifiable with the rs-fMRI technique [18, 41]. For example, Wang T et al. [14] demonstrated that 3 months after CAS patients showed increased regional neural activity of the right precentral gyrus measured with ALFF and increased connectivity of the PCC in the right suprafrontal gyrus. Porcu M et al. [18] detected a reorganization of the brain networks following CEA procedure (3–6 months after the procedure), mostly expressed in terms of increased connectivity between several areas of the brain. This included the medial prefrontal cortex, a pivotal area of the DMN. These rearrangements of the brain activity could also be partly explained by improved myelination of the white matter fibers, as evidenced by Sato Y et al. [41]. The authors further observed using DTI an improvement of the mean values of fractional anisotropy of the white matter located in the hemisphere ipsilateral to surgery and in the contralateral anterior cerebral artery territory. A subsequent connectometry study by Porcu M et al. [42] showed that the short-term changes in interhemispheric local connectivity in the corpus callosum and cerebellum following CEA tend to confirm this hypothesis.

In our research we observed that asymptomatic patients following CEA showed significant improvement of the cognitive performances measured by MMSE. The statistically significant improvement in MMSE scores in the mid-term analysis (12 months) supports the hypothesis that the improvements in cerebral perfusion following the CEA procedure led to better cognitive performances and are in line with the findings from literature [7–10]. Regarding the rs-fMRI analysis, it is noteworthy that our findings are concordant to those found by Wang T et al. [14]. In fact, despite the different number of subjects analyzed, the different scan acquisition modalities, and the different revascularization procedure (CAS versus CEA), our research showed that the right precentral gyrus showed increased regional neural activity following revascularization. Right precentral gyrus is the traditionally implicated in motor control, but according to the recent study of Tomasino B et al. [43], it is probable that this area, as well as the contralateral, is also implicated in higher cognitive tasks (motor imagery, working memory, emotion/empathy, and language), likely as a product of implicit mental simulation processing.

Among the other areas that showed to be more activated following CEA procedure, it is noteworthy to mention the right middle frontal gyrus, which has been proposed to have a role in reorienting attention, working as a circuit-breaker in order to interrupt ongoing endogenous attention processes [44], and both ACC, a pivotal component of the salience network (SN) [45], and PCC, a pivotal component of the DMN [35]. The cingulate cortex is a highly connected and metabolically active brain region [45]. Several studies have suggested that this structure has an important role in cognitive function [45]. The region is typically discussed as having a unitary function because of a common pattern of relative deactivation observed during attentionally demanding tasks [45]. One influential hypothesis is that the PCC has a central role in supporting internally-directed cognition [46]. It has a key node in the DMN and shows increased activity when individuals retrieve autobiographical memories or when they plan future activities, as well as during unconstrained “rest” when activity in the brain is “free-wheeling” [47]. Regarding ACC, several papers have sustained that this region plays a key role in mitigating the competition that arises from two simultaneously active signals [48]. Few papers have demonstrated that ACC is necessary for behavioral flexibility, and they have shown that ACC acts by modulating downstream brain regions such as the dorsal medial striatum, a cerebral region that encode action plans necessary for task completion [49]. Finally, dorsal anterior cingulate cortex (dACC) is a core structure for the governing of cognitive control, and recent studies have shown that interindividual differences in dACC anatomy are associated with corresponding differences in the ability for cognitive control [50].

Understanding cingulate cortex function is likely to be of clinical importance, despite the fact that to the best of our knowledge few neuropsychological researches have analyzed the cognitive consequences of focal lesions on ACC and PCC; however, it has been demonstrated that strokes around the posteromedial cortex produce an amnestic syndrome, which may result in part from damage to both the retrosplenial cortex and PCC [51]. It is a matter of fact that an enormous number of papers described a decrease of function in PCC and ACC, especially in neurodegenerative and neurodevelopmental disease as Parkinson disease, dementia, and autism [52–54]. In our paper instead, we have been able to demonstrate an activation of ACC and PCC linked to a statistically significant improvement of neurocognitive performances after CEA.

Although the partial knowledge of the mechanisms underlying neurocognition limits the interpretation of our results, it is reasonable to conclude that the cognitive improvements observed after CEA, in analogy to what seen for CAS [41], are partly determined by a reassessment of regional neural activity of several brain areas. These findings tend to confirm the trend observed in literature, in particular the hypothesis that the improved cerebral perfusion following revascularization procedures induces a series of rearrangements of brain activity and networking that could explain the observed improvements in cognitive performances [18, 40, 42].

Finally, we also speculate that CEA could influence not only cognition, but also other cerebral functions, such as motor and visual functions. For example, in 2019 Yan J et al. [55] produced a scientific paper about the connection between sight function and carotid revascularization; before this publication, specific changes in visual function before and after CEA were not well understood, but in this paper the authors were able to demonstrate that an improvement in carotid artery and ophthalmic artery blood flow after CEA does indeed enhance subjective and objective assessments of visual function in patients with carotid artery stenosis. Another example in this sense is the recent research by Sato S et al. [56], in which the authors demonstrated that the improvements in gait function observed after CEA are linked with postoperative recovery in perfusion and neurotransmitter receptor function in the motor-related cerebral cortex.

In conclusion, these data could enlighten new opportunity for patient affected by carotid stenosis and cognitive impairment, and might suggest new indication for surgical and endovascular treatment of carotid stenosis. Nonetheless, this was beyond the goal of our study, and future researches are needed to test this hypothesis.

We acknowledge significant limitations in our current study. The first one is the small cohort size; in particular, with this small number of cases we were not allowed to evaluate whether and how confounding factors such as the laterality of the CEA procedure or the variations in the configuration of the circle of Willis influence the ALFF signal. However, our aim was to explore the potential role of ALFF evaluation technique in the study of brain responses after CEA in patient with severe ICA stenosis. The second limitation is the use of MMSE as the only test for the evaluation of the patients’ cognitive function and impairment. However, similar to Grunald IQ et al. [57], even if MMSE is not the sole optimal test for cognitive analysis in patients with ICA stenosis, it was used as preferential test for its ease use in order to give general indications on the trend for neurocognitive performance before and after the surgical procedure, according also to the exploratory nature of the study. Lastly, a potential technical limit could be represented also by the fact that the order of sequence acquisition was not randomized to minimize confounds. Future studies with larger randomized populations, supported by the use of other tests for the analysis of the whole aspects of cognition, included for example the California’s verbal learning test for memory [58] and the trail making test [59] for executive functioning, will be necessary to further expand and enforce the model, and to evaluate the role of revascularization procedures not only for the prevention of stroke, but also for the treatment of neurocognitive deficits associated to ICA stenosis.

## Conclusions

This prospective observational study analyzed the mid-term effects of CEA on neurocognitive status and regional neural activity on rs-fMRI using the ALFF technique in asymptomatic patients with severe ICA stenosis. The results support the hypothesis that the cognitive improvement observed after CEA could be related to increased regional neural activity of several brain area. Our results could represent a starting point to re-think the role of carotid revascularization not only for stroke prevention, but also for treatment of cognitive deficits in selected patients.

## Supplementary Information

Below is the link to the electronic supplementary material.

Supplementary file1 (PDF 115 KB)

## Abbreviations

AAL: Automated Anatomical Labeling
ALFF: Amplitude of Low Frequency Fluctuations
BOLD: Blood oxygen level dependent
CAS: Carotid stenting
CEA: Carotid endarterectomy
DMN: Default mode network
DTI: Diffusion tensor imaging
EPI: Echo-planar imaging
ESC: European Society of Cardiology
GLM: General linear model
ICA: Internal carotid artery
MMSE: Mini-Mental State Examination
MNI: Montreal Neurological Institute
MRI: Magnetic resonance imaging
NASCET: North American Symptomatic Carotid Endarterectomy Trial
p-FDR: *p*-Value corrected for false discovery rate
p-FWE: Family wise corrected *p*-value
p-unc: Uncorrected *p*-value
PCC: Posterior cingulate cortex
rs-fMRI: Resting-state functional magnetic resonance imaging
TFE: Turbo Field Echo
TIA: Transient ischemic attack
