# Supplementary material for: The mid-term effects of carotid endarterectomy on cognition and regional neural activity analyzed with the amplitude of low frequency fluctuations technique
Source: Neuroradiology. 2021 Sep 25;64(3):531–41. doi: 10.1007/s00234-021-02815-7 (PMC8850244; doi:10.1007/s00234-021-02815-7)
Supplement: Supplementary file 1 — Supplementary file1 (PDF 115 KB) [file 234_2021_2815_MOESM1_ESM.pdf]

Supplementary table 1

Atlas – ROIs legends [32,33]

- Atlas: FP r = Frontal Pole Right
- FP l = Frontal Pole Left
- IC r = Insular Cortex Right
- IC l = Insular Cortex Left
- SFG r = Superior Frontal Gyrus Right
- SFG l = Superior Frontal Gyrus Left
- MidFG r = Middle Frontal Gyrus Right
- MidFG l = Middle Frontal Gyrus Left
- IFG tri r = Inferior Frontal Gyrus, pars triangularis Right
- IFG tri l = Inferior Frontal Gyrus, pars triangularis Left
- IFG oper r = Inferior Frontal Gyrus, pars opercularis Right
- IFG oper l = Inferior Frontal Gyrus, pars opercularis Left
- PreCG r = Precentral Gyrus Right
- PreCG l = Precentral Gyrus Left
- TP r = Temporal Pole Right
- TP l = Temporal Pole Left
- aSTG r = Superior Temporal Gyrus, anterior division Right
- aSTG l = Superior Temporal Gyrus, anterior division Left
- pSTG r = Superior Temporal Gyrus, posterior division Right
- pSTG l = Superior Temporal Gyrus, posterior division Left
- aMTG r = Middle Temporal Gyrus, anterior division Right
- aMTG l = Middle Temporal Gyrus, anterior division Left
- pMTG r = Middle Temporal Gyrus, posterior division Right
- pMTG l = Middle Temporal Gyrus, posterior division Left
- toMTG r = Middle Temporal Gyrus, temporooccipital part Right
- toMTG l = Middle Temporal Gyrus, temporooccipital part Left
- aITG r = Inferior Temporal Gyrus, anterior division Right
- aITG l = Inferior Temporal Gyrus, anterior division Left
- pITG r = Inferior Temporal Gyrus, posterior division Right
- pITG l = Inferior Temporal Gyrus, posterior division Left
- toITG r = Inferior Temporal Gyrus, temporooccipital part Right
- toITG l = Inferior Temporal Gyrus, temporooccipital part Left
- PostCG r = Postcentral Gyrus Right
- PostCG l = Postcentral Gyrus Left
- SPL r = Superior Parietal Lobule Right
- SPL l = Superior Parietal Lobule Left
- aSMG r = Supramarginal Gyrus, anterior division Right
- aSMG l = Supramarginal Gyrus, anterior division Left
- pSMG r = Supramarginal Gyrus, posterior division Right
- pSMG l = Supramarginal Gyrus, posterior division Left
- AG r = Angular Gyrus Right
- AG l = Angular Gyrus Left
- sLOC r = Lateral Occipital Cortex, superior division Right
- sLOC l = Lateral Occipital Cortex, superior division Left

- iLOC r = Lateral Occipital Cortex, inferior division Right
- iLOC l = Lateral Occipital Cortex, inferior division Left
- ICC r = Intracalcarine Cortex Right
- ICC l = Intracalcarine Cortex Left
- MedFC = Frontal Medial Cortex
- SMA r = Juxtapositional Lobule Cortex -formerly Supplementary Motor Cortex- Right
- SMA L= Juxtapositional Lobule Cortex -formerly Supplementary Motor Cortex- Left
- SubCalC = Subcallosal Cortex
- PaCiG r = Paracingulate Gyrus Right
- PaCiG l = Paracingulate Gyrus Left
- AC = Cingulate Gyrus, anterior division
- PC = Cingulate Gyrus, posterior division
- Precuneus = Precuneous Cortex
- Cuneal r = Cuneal Cortex Right
- Cuneal l = Cuneal Cortex Left
- FOrb r = Frontal Orbital Cortex Right
- FOrb l = Frontal Orbital Cortex Left
- aPaHC r = Parahippocampal Gyrus, anterior division Right
- aPaHC l = Parahippocampal Gyrus, anterior division Left
- pPaHC r = Parahippocampal Gyrus, posterior division Right
- pPaHC l = Parahippocampal Gyrus, posterior division Left
- LG r = Lingual Gyrus Right
- LG l = Lingual Gyrus Left
- aTFusC r = Temporal Fusiform Cortex, anterior division Right
- aTFusC l = Temporal Fusiform Cortex, anterior division Left
- pTFusC r = Temporal Fusiform Cortex, posterior division Right
- pTFusC l = Temporal Fusiform Cortex, posterior division Left
- TOFusC r = Temporal Occipital Fusiform Cortex Right
- TOFusC l = Temporal Occipital Fusiform Cortex Left
- OFusG r = Occipital Fusiform Gyrus Right
- OFusG l = Occipital Fusiform Gyrus Left
- FO r = Frontal Operculum Cortex Right
- FO l = Frontal Operculum Cortex Left
- CO r = Central Opercular Cortex Right
- CO l = Central Opercular Cortex Left
- PO r = Parietal Operculum Cortex Right
- PO l = Parietal Operculum Cortex Left
- PP r = Planum Polare Right
- PP l = Planum Polare Left
- HG r = Heschl's Gyrus Right
- HG l = Heschl's Gyrus Left
- PT r = Planum Temporale Right
- PT l = Planum Temporale Left
- SCC r = Supracalcarine Cortex Right
- SCC l = Supracalcarine Cortex Left
- OP r = Occipital Pole Right
- OP l = Occipital Pole Left
- Thalamus r = Thalamus Right

- Thalamus l = Thalamus Left
- Caudate r = Caudate Right
- Caudate l = Caudate Left
- Putamen r = Putamen Right
- Putamen l = Putamen Left
- Pallidum r = Pallidum Right
- Pallidum l = Pallidum Left
- Hippocampus r = Hippocampus Right
- Hippocampus l = Hippocampus Left
- Amygdala r = Amygdala Right
- Amygdala l = Amygdala Left
- Accumbens r = Accumbens Right
- Accumbens l = Accumbens Left
- Brain-Stem = Brain Stem
- Cereb1 l = Cerebelum Crus1 Left
- Cereb1 r = Cerebelum Crus1 Right
- Cereb2 l = Cerebelum Crus2 Left
- Cereb2 r = Cerebelum Crus2 Right
- Cereb3 l = Cerebelum 3 Left
- Cereb3 r = Cerebelum 3 Right
- Cereb45 l = Cerebelum 4 5 Left
- Cereb45 r = Cerebelum 4 5 Right
- Cereb6 l = Cerebelum 6 Left
- Cereb6 r = Cerebelum 6 Right
- Cereb7 l = Cerebelum 7b Left
- Cereb7 r = Cerebelum 7b Right
- Cereb8 l = Cerebelum 8 Left
- Cereb8 r = Cerebelum 8 Right
- Cereb9 l = Cerebelum 9 Left
- Cereb9 r = Cerebelum 9 Right
- Cereb10 l = Cerebelum 10 Left
- Cereb10 r = Cerebelum 10 Right
- Ver12 = Vermis 1 2
- Ver3 = Vermis 3
- Ver45 = Vermis 4 5
- Ver6 = Vermis 6
- Ver7 = Vermis 7
- Ver8 = Vermis 8
- Ver9 = Vermis 9
- Ver10 = Vermis 10

Subject	Age	Sex	Number of valid scans	Number of invalid scans	Max motion (mm)	Mean motion (mm)	Maximum global signal change (std)	Mean global signal change (std)	BOLD signal after denoising (std)	Global correlation (Pre-CEA)	Global correlation (Pre-CEA)
1	76	Male	**611**	29	2.37216	0.180842	9.149505	0.856914	0.464154	0.007372	0.015467
2	72	Female	**626**	14	3.113677	0.1581	5.702185	0.858364	0.525145	0.016688	0.024355
3	65	Male	**623**	17	1.81664	0.158935	9.325184	0.791786	0.446014	0.019116	0.016557
4	83	Female	**558**	82	4.882054	0.287191	5.527984	0.813036	0.545063	0.018117	0.015489
5	76	Female	**613**	27	3.653743	0.198421	4.078381	0.812803	0.527557	0.009692	0.008462
6	76	Female	**592**	48	2.098371	0.243444	11.473914	0.866751	0.522655	0.010613	0.0115
7	75	Male	**548**	92	3.11209	0.246553	8.039364	0.839988	0.564153	0.018452	0.011537
8	71	Male	**638**	2	1.330331	0.137933	3.142281	0.789739	0.450972	0.016837	0.007309
9	72	Female	**614**	26	3.221927	0.196437	7.152392	0.889551	0.503291	0.006794	0.012755
10	77	Male	**572**	68	3.261153	0.211337	11.523178	0.823741	0.51082	0.011899	0.025415
11	73	Male	**568**	72	9.953674	0.363952	6.111445	0.79778	0.533049	0.017586	0.014453
12	80	Male	**597**	43	1.796954	0.252225	5.108727	0.830967	0.531292	0.011559	0.011462
13	61	Female	**599**	41	1.70287	0.252648	5.287314	0.857589	0.568297	0.019118	0.021214
14	66	Male	**636**	4	1.524617	0.184501	3.198237	0.796792	0.426874	0.012658	0.010497
15	84	Male	**622**	18	1.486636	0.449961	3.425556	0.768043	0.537357	0.017116	0.013421
16	81	Male	**576**	64	3.474708	0.143141	10.2644	0.796964	0.535611	0.011609	0.009441
17	73	Male	**615**	25	3.428577	0.136713	5.291873	0.844085	0.538697	0.014614	0.014139
18	70	Male	**621**	19	2.349323	0.199265	9.53037	0.822858	0.484479	0.010894	0.011838
19	82	Male	**587**	53	3.96837	0.190688	12.216554	0.798537	0.466255	0.009249	0.008893
20	76	Male	**627**	13	2.057372	0.170605	5.320923	0.871514	0.489848	0.012121	0.01072

Supplementary table 2: Quality control data of the study population following fMRI preprocessing. BOLD: Blood oxygen level depended; std: standard deviations.
